# Breast metastasis from endometrial clear cell carcinoma: A case report and review of the literature

**DOI:** 10.3389/fonc.2022.1070744

**Published:** 2023-01-25

**Authors:** Amadora Li En Choo, Llewellyn Shao-jen Sim, Kesavan Sittampalam, Wei Chong Tan, Amos Zhi En Tay, Ravichandran Nadarajah, Veronique Kiak Mien Tan, Yirong Sim

**Affiliations:** ^1^ Department of General Surgery, Singapore General Hospital, Singapore, Singapore; ^2^ Department of Diagnostic Radiology, Singapore General Hospital, Singapore, Singapore; ^3^ Department of Anatomical Pathology, Singapore General Hospital, Singapore, Singapore; ^4^ Division of Medical Oncology, National Cancer Centre Singapore, Singapore, Singapore; ^5^ Department of Obstetrics and Gynaecology, Singapore General Hospital, Singapore, Singapore; ^6^ Department of Breast Surgery, Division of Surgery and Surgical Oncology, National Cancer Centre Singapore, Singapore, Singapore; ^7^ Department of Breast Surgery, Division of Surgery and Surgical Oncology, Singapore General Hospital, Singapore, Singapore; ^8^ SingHealth Duke-NUS Breast Centre, Singapore, Singapore

**Keywords:** breast cancer, endometrial cancer, endometrial clear cell carcinoma, metastasis, secondary breast cancer

## Abstract

Metastasis to the breast from extra-mammary malignancies are rare, accounting for less than 1% of all breast cancers. Endometrial cancer, a common gynecological malignancy, often spreads to the pelvis, abdominal lymph nodes, peritoneum or the lungs. Endometrial metastasis to the breast is extremely rare, and while there have been isolated case reports of endometrial serous carcinoma with breast metastasis, it has not been reported in the case of clear cell carcinoma. We present a rare case of a 70 year old Chinese lady who had a metastatic endometrial clear cell carcinoma with metastasis to the breast, mimicking an inflammatory breast cancer clinically. We reviewed the current literature and describe the challenges in differentiating primary from metastatic breast lesions, as well as clinical, radiological and histopathological features that may help to differentiate the two. Tumour metastasis to the breast *via* lymphatic or hematogenous route can affect their radiological features: the former mimicking inflammatory breast cancer and the latter with features similar to benign breast lesions. Regardless, histological features with immunohistochemical staining is still the gold standard in diagnosing metastatic breast lesions and determining their tissue of origin. Breast metastases from extra-mammary malignancies are uncommon and it is even rarer for endometrial clear cell carcinoma to spread to the breast. Nonetheless, this case highlights the importance of keeping an open mind and engaging a multidisciplinary team for the care of complex patients.

## Introduction

Metastasis to the breast from extra-mammary malignancies are rare, accounting for less than 1% of all breast cancers (1). The more common cancers that spread to the breast include melanomas and hematological malignancies such as leukemias and lymphomas (2). Others such as lung, ovarian, and gastric cancers have been reported (3–5) but are rare.

We present to you a rare case of metastatic spread of clear cell endometrial carcinoma to the breast. Endometrial cancer, most commonly affecting postmenopausal women in their sixth or seventh decade, may spread *via* direct local invasion or lymphatic or hematological route. Typical sites of metastasis include local pelvic recurrence, abdominal lymph nodes, peritoneum, or the lungs (6). Endometrial metastasis to the breast is extremely rare. It has been reported in the case of serous histology; however, to the best of our knowledge, endometrial clear cell carcinoma with breast metastasis has not been reported before.

## Case report

A 70-year-old Chinese woman presented with postmenopausal bleeding and a left breast lump for a month. Examination revealed a 6-cm left retro-areolar mass with nipple retraction and skin erythema that resembles inflammatory breast cancer. An ultrasound pelvis showed a 7.0 × 4.9 × 5.4-cm ill-defined hypoechoic mass in the anterior wall of the uterus, and the endometrium was thickened at 8.7 mm.

**Figure 1:**
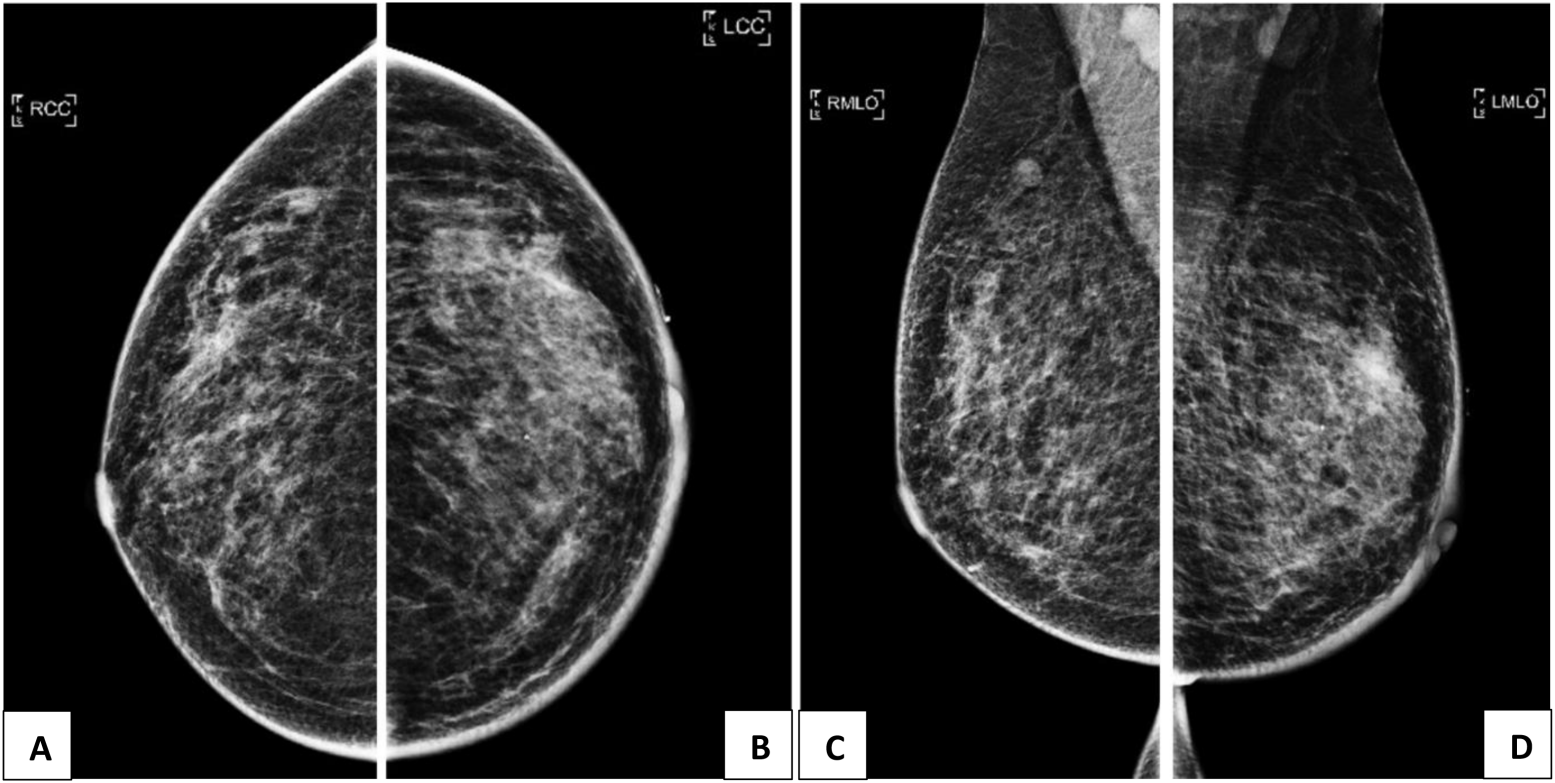
**(A, C)**: Mammogram of the right breast showing an 8 x 8mm partly circumscribed nodule in the right axillary tail, corresponding to the intramammary lymph mode on ultrasound. Abnormal lymph nodes are also seen in bilateral axillae, denser on the right. **(B, D)**: Mammogram of the left breast showing a vague asymmetric density in the upper outer quadrant, corresponding to the palpable lump. The overlying skin is thickened with slight retraction of the nipple.

Mammogram (**Figure 1**) found a vague asymmetric density in the left upper outer quadrant, corresponding to the palpable lump. The overlying skin was thickened with slight retraction of the left nipple. No spiculated mass, suspicious clustered microcalcifications, or architectural distortion was identified. An 8 × 8 mm partially circumscribed nodule was seen in the right axillary tail, corresponding to the intramammary lymph node seen on subsequent ultrasound. Abnormal lymph nodes were seen in both axillae, denser on the right.

**Figure 2:**
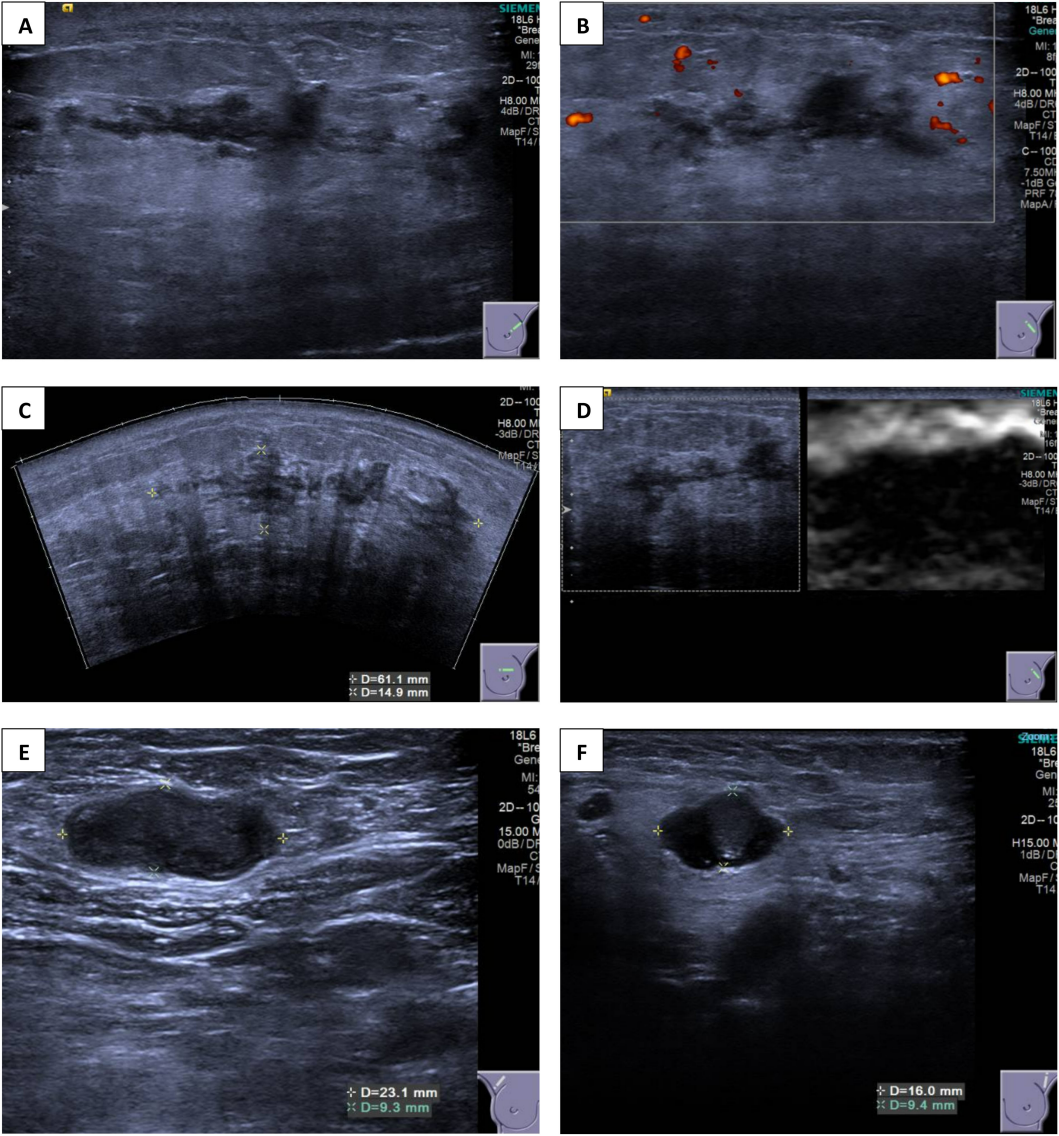
**(A–D)** Ultrasound images of the left breast showing multiple ill-defined heterogeneously hypoechoic lesions extending from the 11 o’clock to 3 o’clock position. **(E, F)** Ultrasound images of bilateral axilla showing abnormal axillary lymphadenopathy.

Breast ultrasound revealed multiple vague and ill-defined heterogeneously hypoechoic lesions extending from the 11 to 3 o’clock positions of the left breast (**Figures 2A–D**). These hypoechoic lesions had irregular margins and were tubular in shape, suggesting an intraductal origin. The whole extent of these lesions measured approximately 61 × 15 × 53 mm (transverse and longitudinal planes) and 42 × 9 × 53 mm (radial and anti-radial planes). The breast stroma surrounding these lesions (and extending to the retroareolar region) appeared echogenic and stiff on elastography, suggesting desmoplasia. This probably accounted for the asymmetric density seen on mammogram. Parenchymal edema and skin thickening were seen in the left breast. In addition, abnormal intramammary lymph nodes were present in the right breast upper outer quadrant, and there were also multiple abnormal lymph nodes in both axillae, compatible with metastasis (**Figures 2E, F**).

The patient underwent concurrent biopsies for both endometrial and breast lesions. She underwent a hysteroscopy, dilatation, and curettage, where the endometrium was found to be irregular and polypoidal with a large tumor that extended to the endocervix. As such, cervical biopsies were also performed to look for cervical involvement or synchronous cervical cancer. Histology from the endometrial curettage revealed high-grade carcinoma with clear cell features, and cervical biopsy revealed endometrial carcinoma.

**Figure 3:**
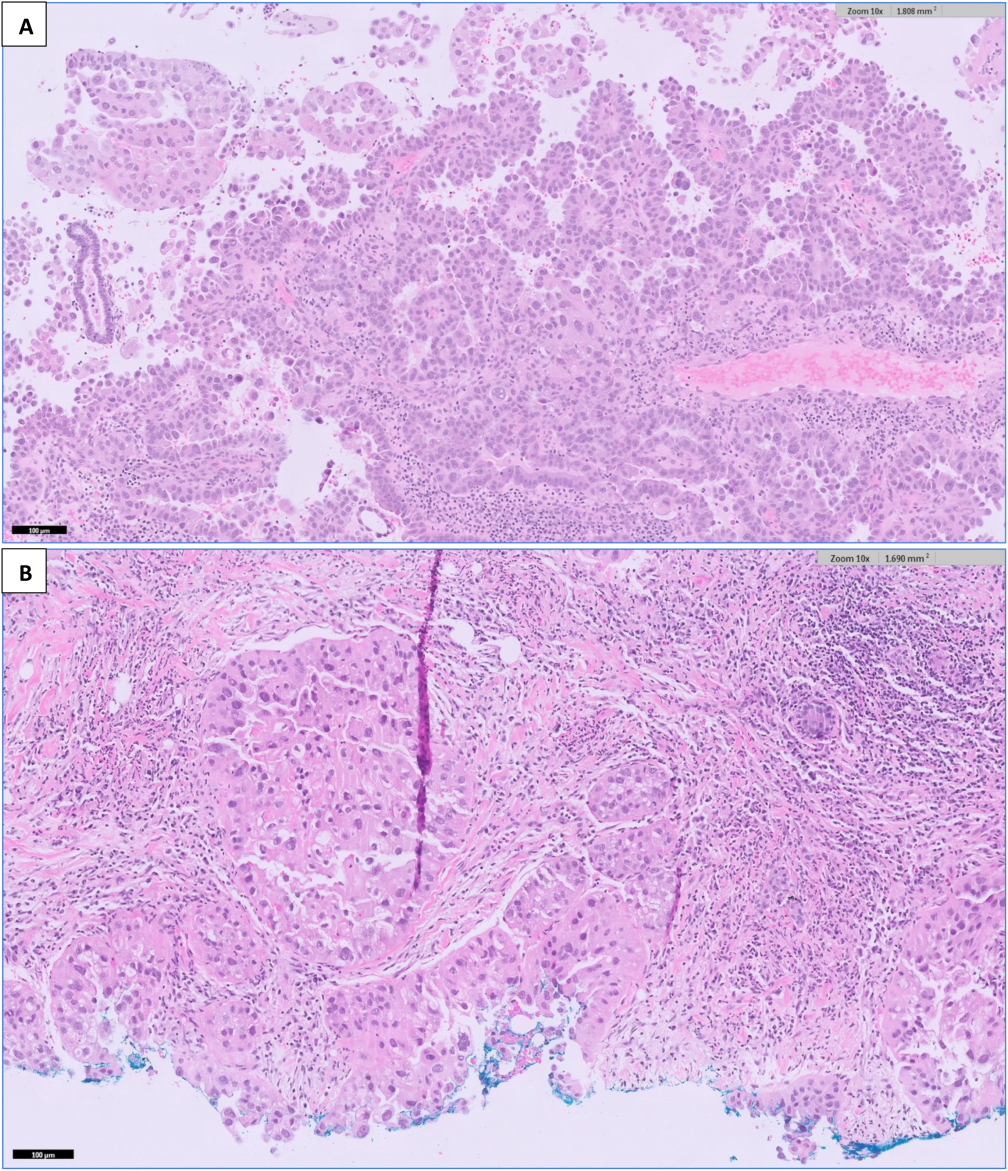
H&E-stained sections at 100× magnification of **(A)** the endometrial curettage specimen, showing high-grade endometrial carcinoma with clear cell features. Fragments of tumor tissue are seen with tubulo-papillary and glandular architecture with a hobnailed appearance. The cells contain nuclear atypia, pleomorphism, and prominent nucleoli with areas of clear cytoplasm. **(B)** The breast tumor shows similar morphology to the endometrial tumor in **Figure 3**.

**Figure 4:**
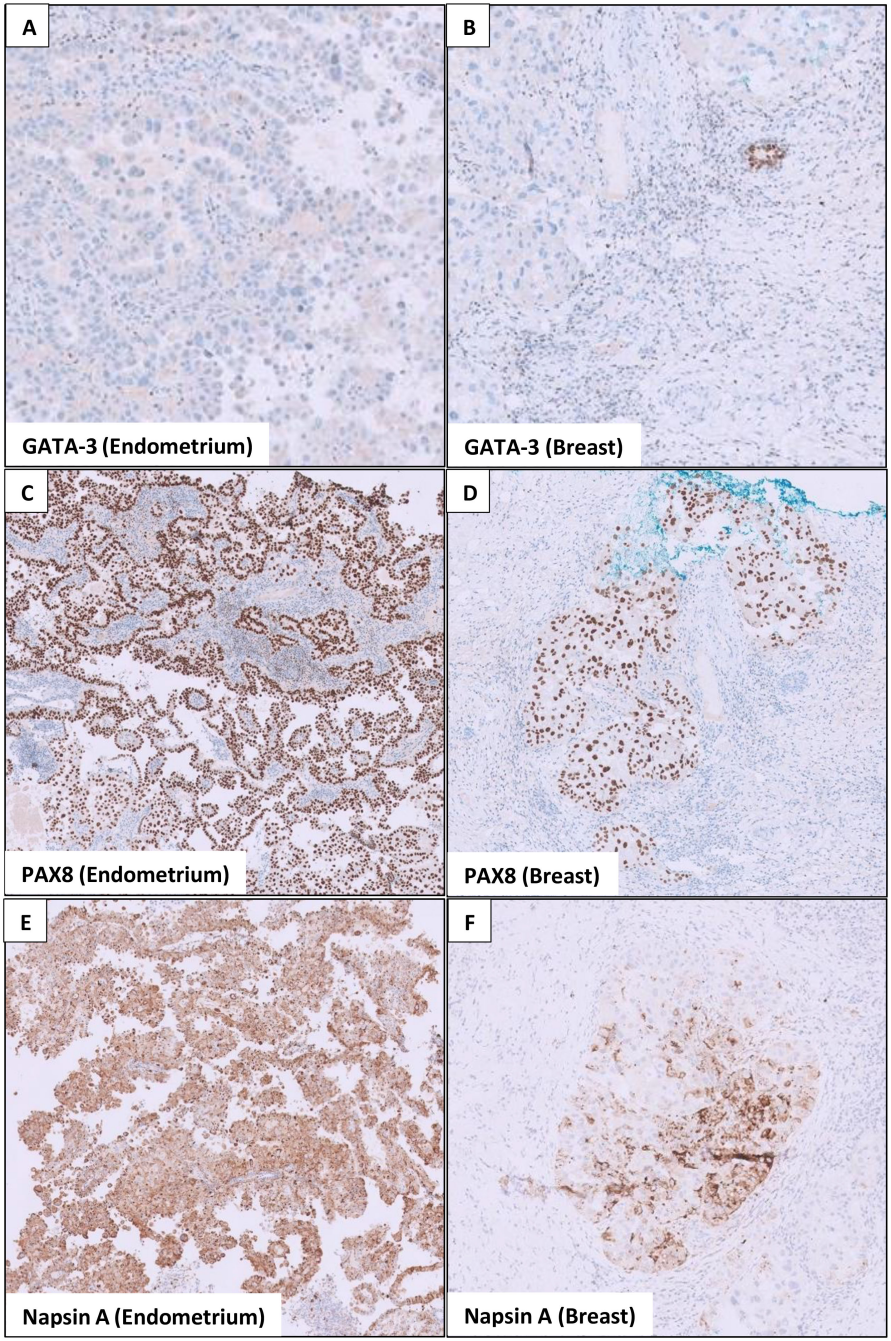
Immunohistochemical stains show similarities in the endometrial and breast tumor. **(A)** The endometrial tumor stains negative for GATA-3 (a breast marker). **(B)** Similarly, the breast tumor also stains negative for GATA-3, although the adjacent breast ducts are positive. **(C, E)** show that the endometrial tumor stains positive for PAX8 (marker for Müllerian tumors) and napsin A (marker for clear cell carcinoma). In **(D, F)** the breast tumor similarly stains positive for PAX8 and napsin A, whereas the adjacent breast tissues are negative.

**Figure 5:**
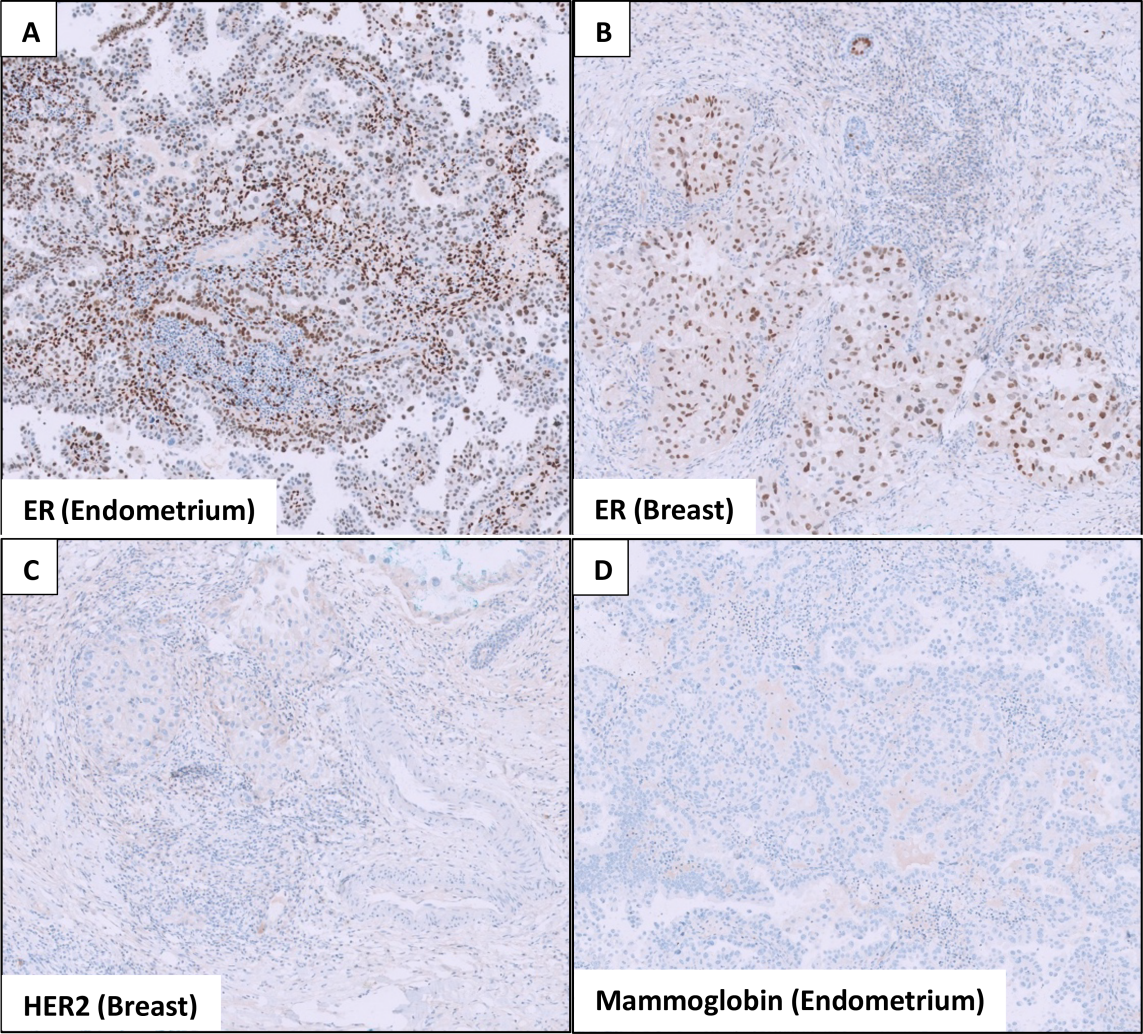
Immunohistochemical stains show that both the **(A)** endometrial and **(B)** breast tumors stain positive for estrogen receptor (ER). **(C)** The breast tumor is negative for HER2 expression, and **(D)** the endometrial tumor does not express mammaglobin, a breast marker.

As for the breast, she underwent ultrasound-guided core needle biopsies of the most prominent ill-defined lesion at the left breast 2 o’clock position and the largest abnormal right axillary lymph node. Interestingly, both the breast and endometrial lesions had significant morphologic resemblance on histology (**Figure 3**). Immunohistochemical staining of the left breast tumor and right axillary lymph node (**Figures 4**, **5**) showed negative results for breast markers GATA-3 and mammaglobin but showed diffusely positive results for PAX8, which is frequently expressed in endometrial carcinoma. We therefore concluded that the left breast lesion and right axillary lymph node were metastasis from the endometrial carcinoma, rather than a breast primary. A positive expression of napsin A supported the diagnosis of a clear cell subtype (7), although it was slightly anomalous in this case that the tumor stained positive for estrogen receptor (ER), which is usually negative or focal in clear cell carcinoma (8). HER2 expression showed a negative result.

A computed tomography scan of the thorax, abdomen, and pelvis showed ascites with omental nodularity concerning for peritoneal metastasis, as well as extensive lymphadenopathy involving the retroperitoneal, pelvic, periportal, bilateral axillary, and mediastinal lymph nodes. Overall findings were suggestive of metastatic endometrial clear cell carcinoma, and the patient was started on palliative chemotherapy with paclitaxel and carboplatin. She was also initiated on letrozole given that her tumor was estrogen receptor (ER)-positive.

## Discussion

Metastatic disease to the breast is rare compared with primary breast cancers. A new lesion in the breast or axilla is far more likely to represent a new primary breast tumor, even in a patient with a history of extramammary malignancy (9). Nonetheless, distinguishing the two is key as treatment and prognosis differ greatly.

Clinically, breast metastasis and primary breast tumors present similarly with a palpable, non-tender lump. However, unlike primary breast cancer, up to a third of patients with extramammary breast metastasis have multiple lesions as opposed to a single solitary tumour (7). Skin changes such as peau d’orange, nipple retraction, and nipple discharge are also uncommon. Notably, metastatic breast masses tend to grow rapidly and close to the skin, possibly due to lymphatic involvement, which may induce skin edema or erythema that may be mistaken for inflammatory breast cancer (10, 11), as was the case for our patient.

Imaging may help to differentiate primary from secondary breast tumors. Features common to primary breast cancer such as spiculations, calcifications, parenchymal distortion, posterior acoustic shadowing, desmoplastic reaction, secondary skin, and nipple changes are typically not seen in breast metastasis (12). Conversely, metastatic lesions tend to be well-circumscribed with clearly defined borders and lack surrounding inflammatory changes, occasionally mimicking benign breast lesions (13, 14). Interestingly, radiological characteristics differ depending on whether the tumor spreads to the breast *via* the lymphatic or hematogenous route. Hematogenous metastasis tends to feature as single or multiple oval, well-circumscribed, and hypoechoic masses affecting the upper outer quadrant, the most richly vascularized area within the breast (13). Axillary lymph node involvement is also less common (14). In contrast, lymphatic spread is often associated with axillary and internal mammary lymphadenopathy. The sonographic appearance of lymphatic metastasis may also mimic inflammatory primary breast cancer by demonstrating heterogenous echogenicity, coarse trabecular pattern, skin thickening, and lymphoedema, due to obstruction of the draining lymphatics (12).

Histopathological examination and immunohistochemistry are key to the diagnosis of breast metastases. The presence of carcinoma *in situ* along with invasive ductal components almost always confirms the diagnosis of primary breast cancer (13). A positive expression of mammaglobin, GATA binding protein 3 (GATA-3), and gross cystic disease fluid protein 15 (GCDFP-15) supports primary breast cancer (15). Triple-negative breast cancers, however, may not express GCDFP-15, GATA 3, or mammaglobin, and in such cases, SOX-10 may be helpful (16). On the other hand, PAX8 expression is a sensitive marker for endometrial carcinoma (17) and napsin A supports the clear cell subtype (18). Nonetheless, it must be recognized that some overlap exists between the immunohistochemical markers seen in breast and endometrial cancers. For example, estrogen receptor (ER), progesterone receptor (PR), and HER2 receptor may be positive or negative in both breast and endometrial cancers (19) and are less useful in distinguishing the two.

In this case, the clinical context, histopathological morphology, and napsin A expression favored the diagnosis of a clear cell subtype of high-grade endometrial carcinoma in the breast. Nonetheless, we acknowledge the diagnostic overlap between serous and clear cell subtypes of high-grade endometrial cancers, compounded by the lack of a definite biomarker to differentiate them. Therefore, it is important to be mindful of the range of high-grade endometrial cancers that may appear in the breast and to consider all differentials. As breast metastases from extramammary malignancies can be challenging to diagnose and manage, a multidisciplinary team involving a breast surgeon, radiologist, pathologist, and medical oncologist is often helpful.

To the best of our knowledge, there have been seven cases of endometrial serous carcinoma with breast metastasis reported in the current literature (9, 12, 20, 21); however, this is the first case report of clear cell endometrial carcinoma with breast metastasis. This is likely because clear cell carcinoma is a rare subtype accounting for less than 5% of endometrial carcinomas. Yet, they are associated with an aggressive clinical behavior and unfavorable prognosis (22). They have an increased propensity for lymphovascular invasion and approximately 45% of patients having extrauterine metastasis at time of diagnosis (23). Microscopically, they are characterized by clear hobnail cells and majority are positive for napsin A, as was the case for our patient.

## Conclusion

Breast metastases from extramammary malignancies are rare. Endometrial clear cell carcinoma metastasizing to the breast is even more unique, with this being the first known case report in the current literature. In general, the prognosis of patients with metastasis to the breast is poor, with a reported median survival of only 10–15 months from the time of diagnosis and over 70% having widespread metastatic disease (13). As such, an early and accurate diagnosis of extramammary breast metastasis is important, so that appropriate treatment can be instituted for these patients.

## Data availability statement

The original contributions presented in the study are included in the article/supplementary material. Further inquiries can be directed to the corresponding author.

## Author contributions

AC and YS conceptualized the manuscript. AC wrote the original draft of the manuscript. LS contributed to the radiology images and assisted with revisions of the manuscript. KS and AT contributed to the histology slides. All authors were involved in the care of the patient and have read and approved the final paper.
